# Supplementary material for: Shaping the physical world to our ends through the left PF technical-cognition area
Source: eLife. 2025 Apr 17;13:RP94578. doi: 10.7554/eLife.94578 (PMC12005713; doi:10.7554/eLife.94578)
Supplement: Supplementary file 1. [file elife-94578-supp1.docx]

| **Table S1. Local maxima of activation clusters (MNI stereotactic coordinates) for the Mechanical problem-solving task (Experimental condition > Control condition).** | | | | | | |
| --- | --- | --- | --- | --- | --- | --- |
| Cluster size | Hemisphere | Brain region | Peak coordinates | | | *t*-value |
|  |  |  | *x* | *y* | *z* |  |
| 1963 | Left | Supramarginal gyrus (PF) | -59 | -30 | 41 | 11.69 |
|  |  | Supramarginal gyrus (PF) | -59 | -32 | 31 | 11.31 |
|  |  | Superior parietal cortex | -8 | -57 | 66 | 11.05 |
| 696 | Left | Dorsal premotor cortex | -20 | -9 | 54 | 14.98 |
|  |  | Dorsal premotor cortex | -18 | 7 | 66 | 10.14 |
|  |  | Dorsal premotor cortex | -18 | -7 | 71 | 9.52 |
| 246 | Left | Inferior frontal gyrus (triangular part) | -47 | 37 | 6 | 9.05 |
|  |  | Anterior prefrontal cortex | -45 | 41 | 20 | 8.86 |
|  |  | Dorsolateral prefrontal cortex | -41 | 37 | 13 | 7.89 |
| 186 | Left | Inferior frontal gyrus (opercular part) | -50 | 7 | 20 | 9.89 |
| 303 | Right | Cerebellum | 26 | -57 | -54 | 9.71 |
|  |  | Cerebellum | 28 | -50 | -26 | 8.96 |
|  |  | Cerebellum | 40 | -48 | -35 | 8.64 |
| These results are also illustrated in Figure 2A. PF, parietal area F. | | | | | | |
