# Supplementary material for: Shaping the physical world to our ends through the left PF technical-cognition area
Source: eLife. 2025 Apr 17;13:RP94578. doi: 10.7554/eLife.94578 (PMC12005713; doi:10.7554/eLife.94578)
Supplement: Supplementary file 2. [file elife-94578-supp2.docx]

| **Table S2. Local maxima of activation clusters (MNI stereotactic coordinates) for the Psychotechnical task (Experimental condition > Control condition).** | | | | | | |
| --- | --- | --- | --- | --- | --- | --- |
| Cluster size | Hemisphere | Brain region | Peak coordinates | | | *t*-value |
|  |  |  | *x* | *y* | *z* |  |
| 1196 | Left | Supramarginal gyrus (PF) | -57 | -32 | 38 | 13.94 |
|  |  | Superior parietal cortex | -20 | -69 | 48 | 10.49 |
|  |  | Intraparietal sulcus | -38 | -34 | 41 | 10.31 |
| 783 | Left | Lateral occipitotemporal cortex | -50 | -66 | -1 | 14.54 |
|  |  | Lateral occipitotemporal cortex | -41 | -66 | -1 | 11.28 |
|  |  | Lateral occipitotemporal cortex | -43 | -44 | -15 | 8.16 |
| 216 | Left | Dorsal premotor Cortex | -22 | -7 | 54 | 11.59 |
| 213 | Left | Inferior frontal gyrus (opercular part) | -50 | 7 | 27 | 8.50 |
| 2629 | Right | Superior parietal cortex | 24 | -62 | 50 | 12.4 |
|  |  | Lateral occipitotemporal cortex | 44 | -62 | -8 | 11.62 |
|  |  | Intraparietal sulcus | 37 | -39 | 45 | 11.23 |
| 298 | Right | Inferior frontal gyrus (opercular part) | 49 | 9 | 27 | 9.77 |
|  |  | Inferior frontal gyrus (opercular part) | 49 | 9 | 18 | 8.50 |
| 230 | Right | Dorsal premotor cortex | 24 | 0 | 52 | 8.65 |
|  |  | Dorsal premotor cortex | 30 | 2 | 66 | 7.49 |
| These results are also illustrated in Figure 2B. PF, parietal area F. | | | | | | |
