# Supplementary material for: Shaping the physical world to our ends through the left PF technical-cognition area
Source: eLife. 2025 Apr 17;13:RP94578. doi: 10.7554/eLife.94578 (PMC12005713; doi:10.7554/eLife.94578)
Supplement: Supplementary file 3. [file elife-94578-supp3.docx]

| **Table S3. Local maxima of activation clusters (MNI stereotactic coordinates) for the Fluid-cognition task (Experimental condition > Control condition).** | | | | | | |
| --- | --- | --- | --- | --- | --- | --- |
| Cluster size | Hemisphere | Brain region | Peak coordinates | | | *t*-value |
|  |  |  | *x* | *y* | *z* |  |
| 1147 | Left | Dorsal premotor cortex | -29 | 0 | 59 | 11.64 |
|  |  | Dorsolateral prefrontal cortex | -47 | 25 | 29 | 10.67 |
|  |  | Inferior frontal gyrus (opercular part) | -45 | 7 | 31 | 10.08 |
| 127 | Left | Thalamus | -13 | -25 | 13 | 8.99 |
|  |  | Thalamus | -6 | -12 | 6 | 7.86 |
|  |  | Thalamus | -20 | -30 | 4 | 7.85 |
| 140 | Left | Insula | -31 | 23 | 2 | 12.36 |
| 974 | Right | Inferior frontal gyrus (opercular part) | 40 | 11 | 31 | 11.95 |
|  |  | Inferior frontal gyrus (opercular part) | 49 | 21 | 31 | 10.90 |
|  |  | Inferior frontal gyrus | 46 | 7 | 31 | 10.15 |
| 661 | Right | Dorsal premotor cortex | 30 | 2 | 57 | 13.25 |
|  |  | Dorsal prefrontal cortex | 30 | 11 | 50 | 9.73 |
|  |  | Dorsal prefrontal cortex | 24 | 21 | 50 | 9.04 |
| 201 | Right | Insula | 33 | 21 | 4 | 13.63 |
| 9887 | Left/Right | Cerebellum | -8 | -73 | -24 | 16.08 |
|  |  | Cerebellum | 3 | -71 | -24 | 15.84 |
|  |  | Occipital cortex | -38 | -76 | -12 | 14.88 |
| 806 | Left/Right | Medial superior frontal cortex | -6 | 11 | 52 | 11.49 |
|  |  | Medial superior frontal cortex | 8 | 16 | 45 | 10.77 |
|  |  | Medial superior frontal cortex | -6 | 27 | 43 | 9.82 |
| 644 | Left/Right | Thalamus | 5 | -25 | -5 | 13.45 |
|  |  | Thalamus | -4 | -28 | -5 | 12.63 |
|  |  | Thalamus | 10 | -23 | 13 | 9.94 |
| These results are also illustrated in Figure 2C. Figure 2C shows activation in the superior parietal cortices (SPC) and lateral occipitotemporal cortices (LOTC), which could seem not to be reported here. This activation belongs to the cluster with the size of 9887, which is a very large cluster. | | | | | | |
