# Supplementary material for: Shaping the physical world to our ends through the left PF technical-cognition area
Source: eLife. 2025 Apr 17;13:RP94578. doi: 10.7554/eLife.94578 (PMC12005713; doi:10.7554/eLife.94578)
Supplement: Supplementary file 4. [file elife-94578-supp4.docx]

| **Table S4. Local maxima of activation clusters (MNI stereotactic coordinates) for the Mentalizing task (PHYS-Only condition > Control condition).** | | | | | | |
| --- | --- | --- | --- | --- | --- | --- |
| Cluster size | Hemisphere | Brain region | Peak coordinates | | | *t*-value |
|  |  |  | *x* | *y* | *z* |  |
| 274 | Left | Lateral occipitotemporal cortex | -50 | -62 | 4 | 10.27 |
|  |  | Lateral occipitotemporal cortex | -54 | -66 | -5 | 8.91 |
|  |  | Lateral occipitotemporal cortex | -45 | -69 | -1 | 8.75 |
| 133 | Left | Supramarginal gyrus (PF) | -59 | -25 | 34 | 8.44 |
|  |  | Supramarginal gyrus (PF) | -57 | -34 | 41 | 7.97 |
|  |  | Supramarginal gyrus (PF) | -52 | -37 | 34 | 7.90 |
| 267 | Right | Lateral occipitotemporal cortex | 46 | -60 | -3 | 9.97 |
|  |  | Lateral occipitotemporal cortex | 51 | -53 | -1 | 8.22 |
|  |  | Lateral occipitotemporal cortex | 49 | -64 | 6 | 6.63 |
| 138 | Right | Supramarginal gyrus | 60 | -28 | 48 | 7.96 |
|  |  | Supramarginal gyrus | 58 | -23 | 34 | 7.62 |
|  |  | Supramarginal gyrus | 51 | -32 | 50 | 6.70 |
| These results are also illustrated in Figure 2D. PF, parietal area F. | | | | | | |
