# Supplementary material for: Shaping the physical world to our ends through the left PF technical-cognition area
Source: eLife. 2025 Apr 17;13:RP94578. doi: 10.7554/eLife.94578 (PMC12005713; doi:10.7554/eLife.94578)
Supplement: Supplementary file 5. [file elife-94578-supp5.docx]

| **Table S5. Local maxima of activation clusters (MNI stereotactic coordinates) for the Mentalizing task (INT+PHYS condition > Control condition).** | | | | | | |
| --- | --- | --- | --- | --- | --- | --- |
| Cluster size | Hemisphere | Brain region | Peak coordinates | | | *t*-value |
|  |  |  | *x* | *y* | *z* |  |
| 1464 | Left | Lateral occipitotemporal cortex | -50 | -71 | -3 | 13.28 |
|  |  | Lateral occipitotemporal cortex | -43 | -46 | -21 | 10.72 |
|  |  | Angular gyrus | -54 | -64 | 8 | 10.59 |
| 256 | Left | Cerebellum | -18 | -71 | -28 | 10.48 |
|  |  | Cerebellum | -15 | -78 | -42 | 9.57 |
| 122 | Left | Supramarginal gyrus (PF) | -59 | -32 | 31 | 9.02 |
| 1752 | Right | Lateral occipitotemporal cortex | 46 | -60 | 2 | 14.35 |
|  |  | Lateral occipitotemporal cortex | 51 | -64 | -5 | 11.72 |
|  |  | Angular gyrus | 42 | -55 | 15 | 11.13 |
| 282 | Right | Temporal pole | 51 | 2 | -24 | 9.60 |
|  |  | Middle temporal gyrus | 51 | -5 | -19 | 9.13 |
|  |  | Temporal pole | 49 | 18 | -28 | 8.74 |
| 165 | Right | Inferior frontal gyrus (triangular part) | 51 | 30 | 8 | 8.42 |
|  |  | Inferior frontal gyrus (opercular part) | 44 | 7 | 20 | 7.28 |
|  |  | Inferior frontal gyrus (opercular part) | 51 | 18 | 25 | 6.23 |
| These results are also illustrated in Figure 2E. PF, parietal area F. | | | | | | |
