# Supplementary material for: Shaping the physical world to our ends through the left PF technical-cognition area
Source: eLife. 2025 Apr 17;13:RP94578. doi: 10.7554/eLife.94578 (PMC12005713; doi:10.7554/eLife.94578)
Supplement: Supplementary file 6. [file elife-94578-supp6.docx]

| **Table S6. Local maxima of activation clusters (MNI stereotactic coordinates) for the Mentalizing task (INT+PHYS condition > PHYS-Only condition).** | | | | | | |
| --- | --- | --- | --- | --- | --- | --- |
| Cluster size | Hemisphere | Brain region | Peak coordinates | | | *t-*value |
|  |  |  | *x* | *y* | *z* |  |
| 235 | Left | Angular gyrus | -50 | -62 | 25 | 8.56 |
|  |  | Angular gyrus | -43 | -57 | 27 | 7.83 |
|  |  | Angular gyrus | -57 | -50 | 13 | 7.66 |
| 310 | Right | Angular gyrus | 49 | -50 | 18 | 9.15 |
|  |  | Lateral occipitotemporal cortex | 51 | -60 | 15 | 8.99 |
|  |  | Lateral occipitotemporal cortex | 42 | -50 | 13 | 7.71 |
| 212 | Right | Middle temporal gyrus | 53 | -2 | -17 | 8.45 |
|  |  | Temporal pole | 56 | 14 | -26 | 7.39 |
|  |  | Temporal pole | 51 | 7 | -28 | 7.32 |
| 163 | Left/Right | Medial prefrontal cortex | -4 | 53 | 29 | 8.22 |
|  |  | Medial prefrontal cortex | 3 | 53 | 20 | 7.76 |
| These results are also illustrated in Figure 2F. | | | | | | |
