# Supplementary material for: Shaping the physical world to our ends through the left PF technical-cognition area
Source: eLife. 2025 Apr 17;13:RP94578. doi: 10.7554/eLife.94578 (PMC12005713; doi:10.7554/eLife.94578)
Supplement: Supplementary file 7. [file elife-94578-supp7.docx]

| **Table S7. Local maxima of activation clusters (MNI stereotactic coordinates) for the conjunction analysis (Mechanical problem-solving AND Psychotechnical AND INT+PHYS AND PHYS-Only).** | | | | | |
| --- | --- | --- | --- | --- | --- |
| Cluster size | Hemisphere | Brain region | Peak coordinates | | |
|  |  |  | *x* | *y* | *z* |
| 374 | Left | Supramarginal gyrus (PF) | -56 | -29 | 36 |
| These results are also illustrated in Fig. 2G. PF, parietal area F. | | | | | |
